# Performance of a screening-trained DL model for pulmonary nodule malignancy estimation of incidental clinical nodules

**DOI:** 10.1007/s00330-025-11829-1

**Published:** 2025-07-15

**Authors:** Renate Dinnessen, Dré Peeters, Noa Antonissen, Firdaus A. A. Mohamed Hoesein, Hester A. Gietema, Ernst Th. Scholten, Cornelia Schaefer-Prokop, Colin Jacobs

**Affiliations:** 1 Department of Medical Imaging, Radboud University Medical Center, Nijmegen, The Netherlands; 2 Department of Radiology, University Medical Center Utrecht, Utrecht University, Utrecht, The Netherlands; 3 Department of Radiology and Nuclear Medicine, Maastricht University Medical Center, Maastricht University, Maastricht, The Netherlands; 4 Maastricht University, GROW, School of Oncology and Reproduction, Maastricht, The Netherlands; 5 Department of Radiology, Meander Medical Center, Amersfoort, The Netherlands

**Keywords:** Lung, Neoplasms, Tomography (X-ray computed), Artificial intelligence, Solitary pulmonary nodule

## Abstract

**Objective:**

To test the performance of a DL model developed and validated for screen-detected pulmonary nodules on incidental nodules detected in a clinical setting.

**Materials and methods:**

A retrospective dataset of incidental pulmonary nodules sized 5–15 mm was collected, and a subset of size-matched solid nodules was selected. The performance of the DL model was compared to the Brock model. AUCs with 95% CIs were compared using the DeLong method. Sensitivity and specificity were determined at various thresholds, using a 10% threshold for the Brock model as reference. The model’s calibration was visually assessed.

**Results:**

The dataset included 49 malignant and 359 benign solid or part-solid nodules, and the size-matched dataset included 47 malignant and 47 benign solid nodules. In the complete dataset, AUCs [95% CI] were 0.89 [0.85, 0.93] for the DL model and 0.86 [0.81, 0.92] for the Brock model (*p* = 0.27). In the size-matched subset, AUCs of the DL and Brock models were 0.78 [0.69, 0.88] and 0.58 [0.46, 0.69] (*p* < 0.01), respectively. At a 10% threshold, the Brock model had a sensitivity of 0.49 [0.35, 0.63] and a specificity of 0.92 [0.89, 0.94]. At a threshold of 17%, the DL model matched the specificity of the Brock model at the 10% threshold, but had a higher sensitivity (0.57 [0.43, 0.71]). Calibration analysis revealed that the DL model overestimated the malignancy probability.

**Conclusion:**

The DL model demonstrated good discriminatory performance in a dataset of incidental nodules and outperformed the Brock model, but may need recalibration for clinical practice.

**Key Points:**

***Question*** *What is the performance of a DL model for pulmonary nodule malignancy risk estimation developed on screening data in a dataset of incidentally detected nodules*?

***Findings*** *The DL model performed well on a dataset of nodules from clinical routine care and outperformed the Brock model in a size-matched subset*.

***Clinical relevance*** *This study provides further evidence about the potential of DL models for risk stratification of incidental nodules, which may improve nodule management in routine clinical practice*.

**Graphical Abstract:**

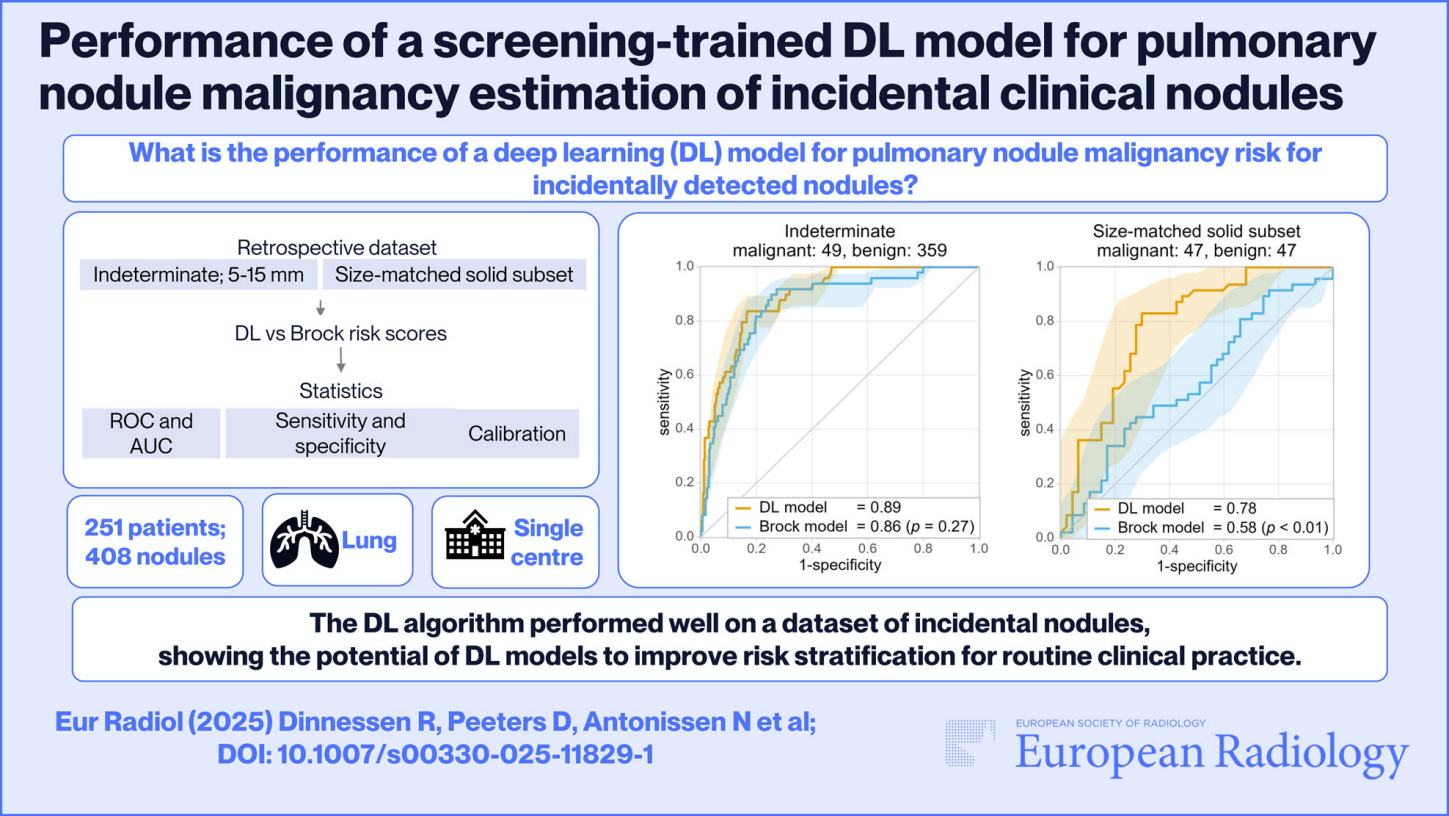

## Introduction

The increased use of computed tomography (CT) has led to a rise in incidentally detected pulmonary nodules [1, 2]. While most are benign, some represent early-stage lung cancer [1, 2]. As lung cancer is often diagnosed late and remains the leading cause of cancer-related deaths [3], early detection is crucial. Randomised trials show that annual low-dose CT screening in high-risk patients reduces lung cancer mortality by 20–24% [4, 5]. Therefore, early detection of nodules may offer a sizeable opportunity to drive a stage shift, even outside lung cancer screening programs. However, distinguishing malignant and benign nodules remains a major clinical challenge.

Pulmonary nodule management guidelines recommend follow-up based on imaging features such as size, morphology, location, and growth [6, 7]. Size and growth are often the main factors guiding decision-making, requiring follow-up imaging. Malignancy risk calculators also incorporate pulmonary comorbidities and demographics [8, 9]. The Brock model is one such tool, developed using data from the Pan-Canadian Early Detection of Lung Cancer Study, and incorporated into the British Thoracic Society (BTS) guidelines for pulmonary nodules (BTS) [6, 8]. Improving nodule classification could reduce unnecessary additional imaging, thereby decreasing the radiologist’s workload, patient’s radiation exposure, and the healthcare costs.

Most research on lung nodule characterisation using Artificial Intelligence (AI has been conducted using lung cancer screening data, where deep learning (DL) models have demonstrated high sensitivity and specificity [10, 11]. However, it is uncertain how applicable these models are for clinical routine care data, as the patient population and CT acquisition in clinical routine care are more diverse, and risk profiles differ. Promising results on data from clinical routine care have been published, but research thus far has been limited [12].

For this study, we used an in-house developed DL model, trained and validated on data from lung cancer screening [11]. The DL model showed equal performance to expert thoracic radiologists and significantly better performance than the clinically established Brock model. We assessed the performance of our in-house DL model for malignancy risk estimation on clinical routine care data and compared it to the Brock model. Secondly, we assessed the calibration of the DL model in clinical routine care data, aiming to evaluate whether the predictions from the DL model are directly translatable to a more diverse clinical cohort.

## Methods

### Study design and population

The Medical Ethical Committee waived the need for informed consent, as this study was a retrospective study using pseudonymised data.

We used a dataset previously described by Hendrix et al [1]. In short, a list of all adult patients who received a chest CT at a Dutch University Medical Centre between 2000 and 2019 was collected and sent to the Netherlands Comprehensive Cancer Organization. A list of any cancer diagnoses for these patients documented by the Netherlands Cancer Registry (NCR) was obtained in 2021. The NCR documents all cancers diagnosed within the Netherlands and has a coverage of 95% [13]. CT scans, including metadata, were retrieved from the Picture Archiving and Communication System, and radiology reports from the Electronic Health Records.

For this retrospective validation study, we aimed to collect a dataset of solid and part-solid incidental nodules sized 5–15 mm, deemed indeterminate by current management guidelines and therefore requiring CT follow-up for characterisation. Participants with malignant lesions were included when they received a stage 1 lung cancer diagnosis (International Classification of Diseases (10th Revision) codes C341, C342, C343, C348, or C349) between 2010 and 2019. Participants with benign lesions were selected from 2016 to 2017 and included if they had no cancer diagnosis documented by the NCR.

For this study, we selected patients aged 18 years and older with nodules sized 5–15 mm. At least two years of follow-up or histological confirmation were required for nodule characterisation. Patient and nodule eligibility criteria were established to exclude patients in a different diagnostic pathway (i.e. patients with any cancer diagnosis before the CT scan, MEN1 syndrome, benign nodular diseases (e.g. rheumatoid arthritis, common variable immunodeficiency, granulomatosis with polyangiitis), and patients with more than ten nodules) or clearly benign nodules (i.e. tree-in-bud configuration, calcified nodules, perifissural opacities, and hamartomas containing macroscopic fatty areas). Furthermore, patients were excluded if they had advanced fibrosis, a nodule diagnosed as carcinoid, or any nodule larger than 15 mm.

Only CT scans with a reconstruction matrix of 512 × 512 or 1024 × 1024 and a slice thickness of 3 mm or less were included. CT scans for planning or controlling a thoracic intervention were excluded. No further eligibility criteria were applied to reflect the heterogeneity of the clinical setting.

### Annotation process

To collect the set of malignant nodules, all patients with a stage 1 lung cancer diagnosis between 2010 and 2019 were included. CT scans up to two years before the lung cancer diagnosis date were collected; the incidence date and tumour data (i.e. location, morphological type, and stage) were documented. An experienced radiologist (> 20 years of experience in reading chest CTs) was asked to annotate the tumour nodules with access to all previously described information. If the annotating radiologist could not locate the malignant nodule, the patient was excluded.

To collect the set of benign nodules, radiology reports from 2016 and 2017 were analysed using an in-house natural language processing (NLP) algorithm [1]. This NLP algorithm searches a radiology report for any mention of a nodule and has a sensitivity of 94% and a specificity of 96%. Patients were selected when a nodule was mentioned in the conclusion section of the radiology report. CT scans and their respective radiology reports up to four years before and two years after the selected report were retrieved. The radiologist was asked to assess eligibility according to the previously described criteria and to annotate nodules sized 5 mm or larger at first occurrence and the first follow-up CT scan performed at least 3 months later. The radiologist was asked to distinguish between the nodules that require any further action (nodules of interest) and any other nodules in a patient, but was instructed to annotate all nodules. For all nodules of interest, the radiologist annotated the lobe location.

### Malignancy risk estimation models

Malignancy risk scores were estimated using a DL-based malignancy risk estimation algorithm. The DL model was trained on a large cohort of nodules from the National Lung Screening Trial (16,077 nodules, 1249 malignant) [4, 11]. The DL model uses a block of the CT scan of 5 × 5 × 5 cm centred around the nodule. It inputs 2D and 3D samples of the CT scan to estimate the malignancy probability. Details on the development of the model are described elsewhere [11]. This algorithm has been made available for research purposes (https://grand-challenge.org/algorithms/pulmonary-nodule-malignancy-prediction).

The Brock 2a model was used as a comparison [8]. This model excludes the spiculation variable, as spiculation was not annotated in our dataset. We decided not to invite a reader to score spiculation, as spiculation has a high inter-reader variability [14].

Both models output a malignancy risk score ranging from 0% to 100%.

### Statistical analysis

Baseline characteristics of the patients and nodules were compared using the one-way Student’s *t*-test (normally distributed) or Kruskal–Wallis Rank Sum test (non-normally distributed) for continuous variables and Chi-square test (> 5 observations per category) or Fisher-exact test (≤ 5 observations in any category) for categorical variables.

A subset consisting of all solid malignant nodules and size-matched solid benign nodules was selected to test the performance of the models in a more challenging dataset. The case-control ratio was chosen to ensure the largest possible sample size with statistically comparable nodule sizes, resulting in a case-control ratio of 1:1. The comparability of the nodule sizes was assessed using the Kruskal–Wallis Rank Sum test.

To compare the performance of the DL and Brock models, receiver operating characteristic (ROC) curves, area under the ROC curve (AUC), and sensitivity and specificity were used. The 95% confidence intervals (CI) of the ROCs were estimated using bootstrapping with 10,000 samples and the 95% CIs of the AUC using the DeLong method. AUCs were compared using the DeLong method.

Sensitivity and specificity were assessed at risk thresholds ranging from 0% to 20%, focusing on the threshold of 10% as recommended for the Brock model within the BTS guidelines [6]. To allow for direct comparison, we calculated the sensitivity of the DL model at the threshold where the specificity of the DL model matched the specificity of the Brock model at a 10% threshold.

Model calibration on our clinical dataset was assessed using a calibration plot. A calibration plot describes how well the model’s predicted risk scores match the actual outcomes observed in the dataset. In a well-calibrated model, a malignancy risk prediction of 10% means that 10 out of 100 nodules are malignant. If the model calibration is suboptimal, a prediction of 10% risk would mean that the number of actual malignant nodules deviates from 10. A calibration plot was constructed using ten uniformly distributed bins. The average observed outcomes were plotted over the average predicted malignancy probabilities. In a perfect prediction model, the predicted and actual probabilities are equal, resulting in a diagonal line.

The incidence within a dataset influences the calibration plot; thus, we simulated the incidence observed in the target population. The incidence was estimated using the raw data of a study by Hendrix et al [1], which analysed data from two Dutch hospitals, one of which provided the current study’s dataset. The incidence of stage 1 lung cancer between 2010 and 2017 among nodules sized 5–15 mm was 1.1% (68 cancers; 6301 nodules, see Appendix Table A1). To simulate this incidence, all malignant and benign nodules were included, followed by an iterative random reselection of benign nodules from the dataset presented in the current study until an incidence of 1.1% was reached. This method leads to a benign nodule potentially appearing multiple times in the simulated dataset.

Additionally, the correlation between nodule size and the DL and Brock risk scores was assessed using scatter plots and Spearman’s correlation coefficients.

Statistical analyses were performed in R (version 4.0.0) using the libraries ‘tableone’ (version 0.13.2) and ‘pROC’ (version 1.18.5) for the ROC analyses and sensitivity and specificity analyses, and in Python using ‘scikit-learn’ (version 1.3.0) for the calibration plot. *p* values below 0.05 were considered statistically significant.

## Results

**Fig. 1:**
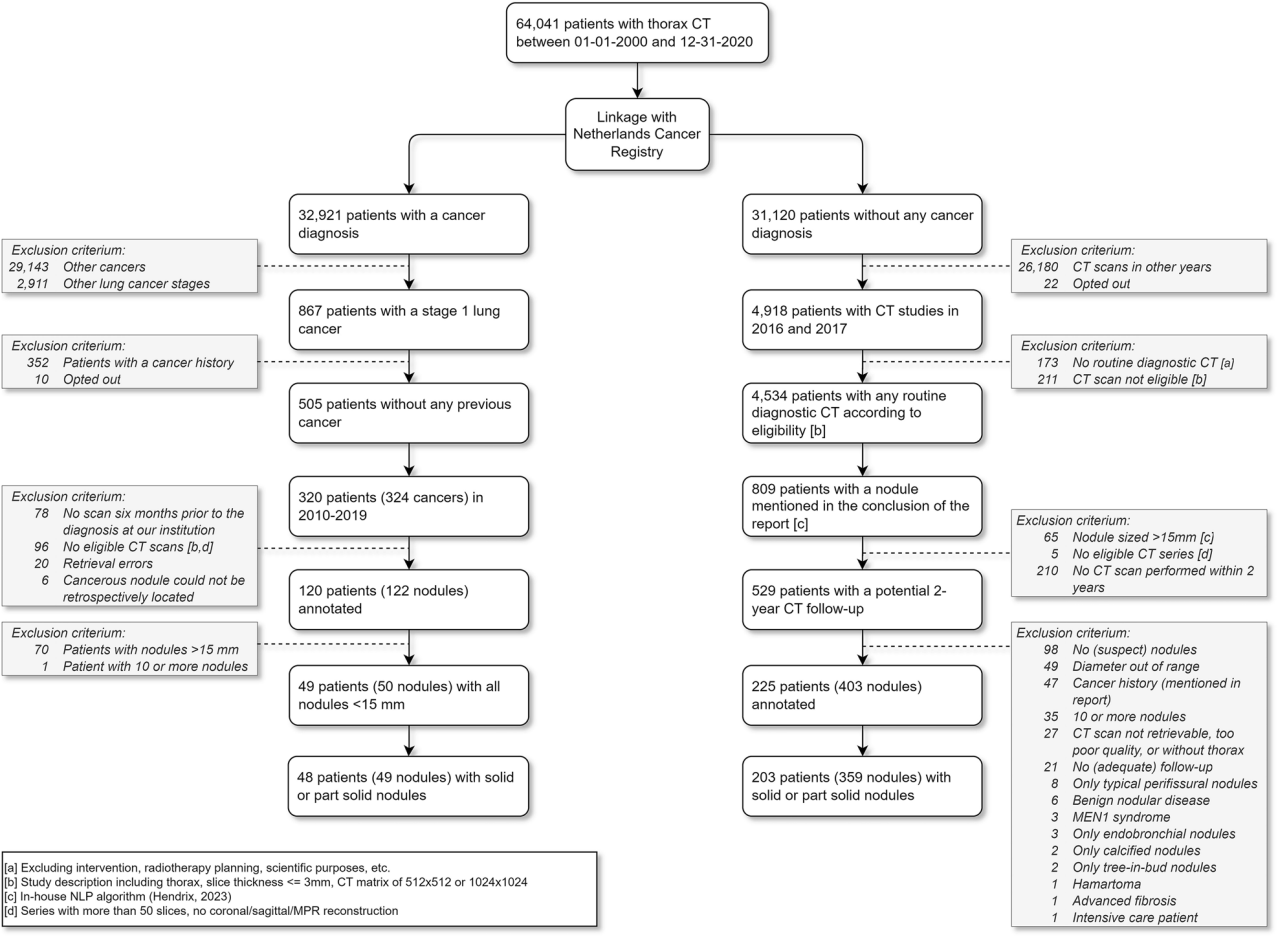
Selection of nodules included in the complete dataset

We collected 359 benign nodules in 203 patients and 49 malignant nodules in 48 patients. The inclusion process is shown in Fig. 1. Patient and nodule characteristics are shown for the complete dataset and the size-matched subset in Table 1. Histological details of the malignant nodules are presented in Table 2. CT characteristics are presented in Table 3, and models and convolution kernel details in Appendix Table A2.

**Table 1:** Baseline characteristics

	Complete dataset				Size-matched subset			
Characteristics	Overall (*n* = 408)	Benign (*n* = 359)	Malignant (*n* = 49)	*p*	Overall (*n* = 94)	Benign (*n* = 47)	Malignant (*n* = 47)	*p*
Gender (female)	194 (48)	169 (47)	25 (51)	0.82	47 (50)	23 (49)	24 (51)	1.00
Age	63 [56, 69]	63 [55, 69]	66 [59, 71]	0.05	65 [59, 70]	65 [58, 70]	66 [60, 71]	0.60
Nodule size (mm)	6.6 [5.6, 8.8]	6.4 [5.4, 8.1]	11.4 [8.7, 13.2]	**<** **0.01**	11.2 [8.8, 13.0]	11.2 [8.8, 12.9]	11.4 [8.8, 13.1]	0.96
Nodule type				1.00				N/A
Part-solid	19 (5)	17 (5)	2 (4)		0 (0)	0 (0)	0 (0)	
Solid	389 (95)	342 (95)	47 (96)		47 (100)	47 (100)	47 (100)	
Number of nodules	3 [1, 4]	3 [1, 4]	2 [1, 3]	**0.01**	2 [1, 4]	2 [2, 4]	2 [1, 3]	**0.03**
Lobe (upper)	186 (46)	151 (42)	35 (71)	**<** **0.01**	56 (60)	23 (50)	33 (70)	0.06
Lobe (specified)				**<** **0.01**				0.12
Left lower	88 (22)	81 (23)	7 (14)		17 (18)	10 (21)	7 (15)	
Left upper	81 (20)	69 (19)	12 (25)		21 (22)	11 (23)	10 (21)	
Right lower	94 (23)	88 (25)	6 (12)		15 (16)	11 (238	6 (13)	
Right middle	40 (10)	39 (11)	1 (2)		6 (6)	5 (11)	1 (2)	
Right upper	105 (26)	82 (23)	23 (47)		35 (37)	12 (26)	23 (49)	

Data are the number of nodules with percentages in parentheses or median values with interquartile ranges in brackets. Significant *p* values are presented in bold

*N/A* not applicable

**Table 2:** Histological details of the malignant nodules

Cancer types	Overall
Neuroendocrine tumour	5 (10)
Neoplasm, NOS	11 (22)
NSCLC	33 (67)
Adenocarcinoma	18
Squamous cell carcinoma	11
Other/general	4

Data are the number of CT studies with percentages in parentheses. No small-cell lung cancer was observed in the dataset

**Table 3:** CT scan parameters

CT characteristics	Overall
Protocol	
Standard	123 (49)
HR	50 (20)
low dose	37 (15)
CTA	38 (15)
Other^a^	3 (1)
Matrix	
512 × 512	225 (90)
1024 × 1024	26 (10)
Slice thickness (mm)	
< 0.5	1 (0.4)
0.5	61 (24)
0.5–1.0	20 (8)
1.0	159 (63)
2.0	4 (2)
3.0	6 (2)
Contrast (applied)	135 (54)
Manufacturer	
Toshiba	158 (63)
Siemens	81 (32)
Philips	11 (43)
GE Medical Systems	1 (0.4)

Data are the number of CT studies with percentages in parentheses or median values with interquartile ranges in brackets

^a^ Other include abdominal, spine, and four-phase liver protocols

**Fig. 2:**
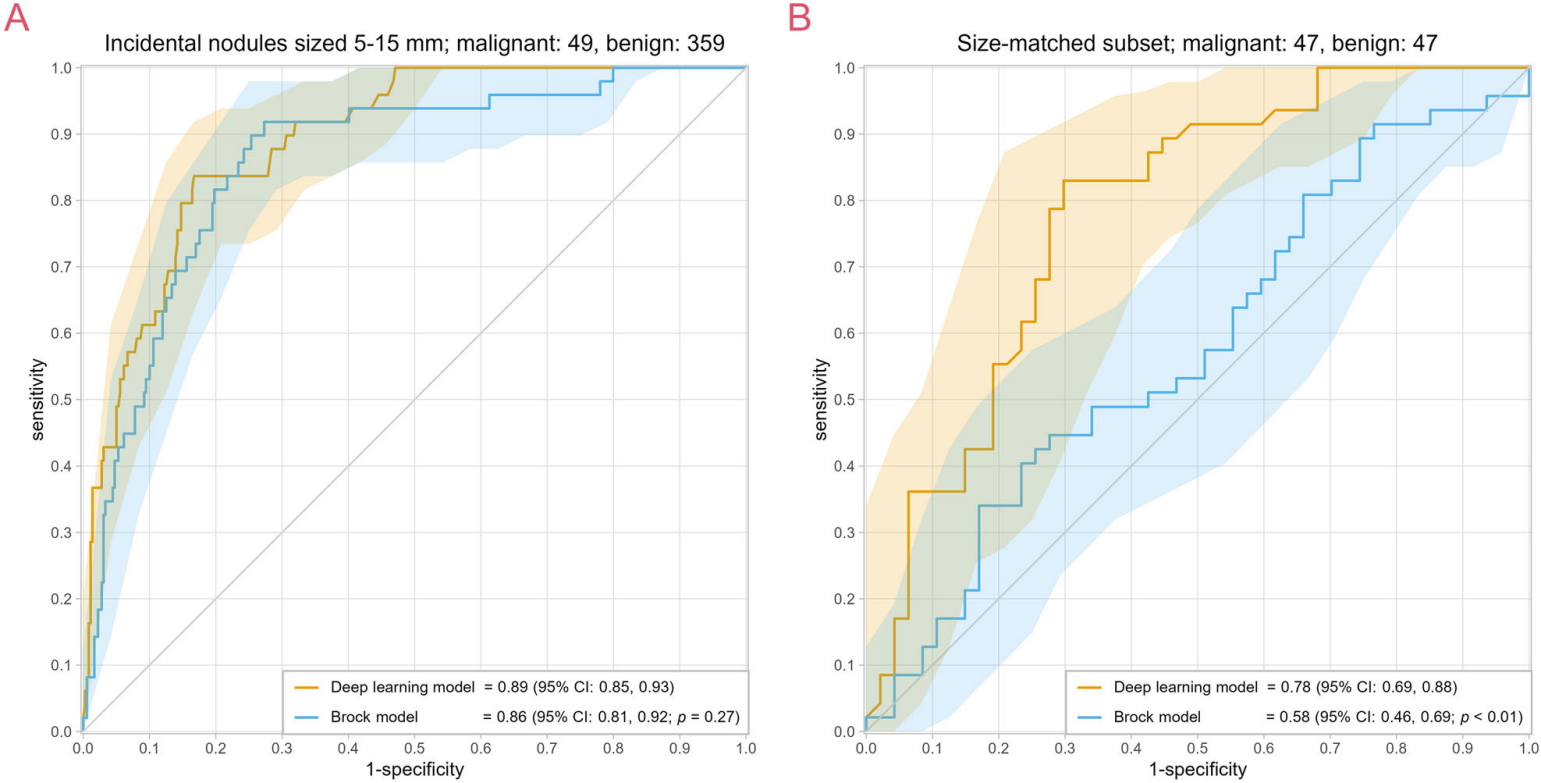
ROC curves with their respective 95% CIs of the DL and Brock models on the (**A**) complete dataset and (**B**) size-matched dataset. The AUC with 95% CIs in parentheses is shown in the legend

Malignant nodules in the complete dataset were on average larger, seen in patients with fewer nodules, and more often located in the upper lobes. In the size-matched subset, the malignant nodules were more often located in the upper lobes, all other variables were not different. Nodule size was not significantly different at a 1:1 case-control ratio, indicating successful size-matching. For the complete dataset, the DL and Brock models achieved equivalent AUCs (0.89; 95% CI: 0.85, 0.93; and 0.86; 95% CI: 0.81, 0.92; *p* = 0.27, respectively). In the size-matched subset, the DL model outperformed the Brock model with an AUC of 0.78 (95% CI: 0.69, 0.88) and 0.58 (95% CI: 0.46, 0.69; *p* < 0.01), respectively. The ROC curves are presented in Fig. 2.

**Fig. 3:**
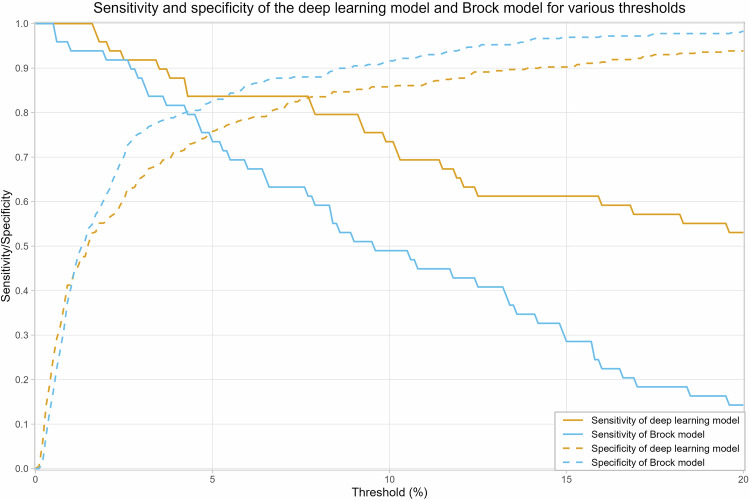
Sensitivity and specificity at various thresholds for the DL and Brock models calculated on the complete dataset

Sensitivities and specificities over thresholds between 0% and 20% are provided in Fig. 3, illustrating that for thresholds of 17–20%, the DL model achieved superior diagnostic performance compared to the Brock model at a 10% threshold. At a threshold of 10%, as recommended for the Brock model by the BTS guidelines to indicate intensified diagnostic workup [6], the Brock model had a sensitivity of 0.49 (95% CI: 0.35, 0.63) and a specificity of 0.92 (95% CI: 0.89, 0.94). The DL model achieved equal specificity at a threshold of 17% as the Brock model at a threshold of 10% but had a higher sensitivity (0.57; 95% CI: 0.43, 0.71). Between thresholds of 17 and 20%, the DL model had a superior sensitivity and specificity to the Brock model.

**Fig. 4:**
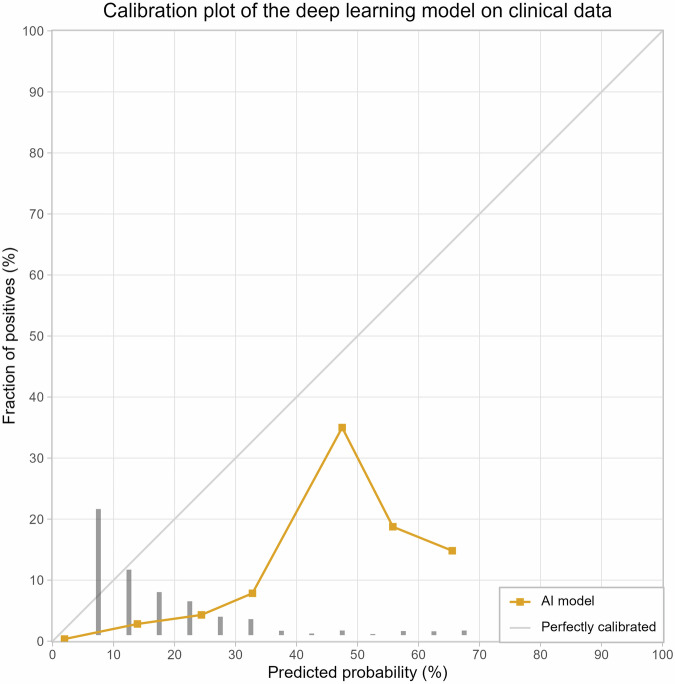
Calibration plot (reliability curves) of the DL model on clinical data, including 4406 benign nodules and 49 malignant nodules. Uniformly distributed buckets of the DL risk score have been used to determine the average predicted probability. The grey bars at the bottom of the figure show the distribution of the DL risk scores for the predicted probabilities shown on the *x*-axis

The calibration plot included a simulated dataset of 4406 benign nodules and 49 malignant nodules and showed that the model overestimated the malignancy probability for all risk scores, see Fig. 4. For example, at an average predicted probability of 25% (third point on the curve in Fig. 4), the actual average probability was 5%. For the predicted probability range from 0% to 35%, the average risk scores increased with the increasing average observed outcomes. The plot shows a peak at a DL score of 45%, but in this bin, only eight nodules were included, the numbers are to be interpreted with caution. Risk scores higher than 70% were not observed for this dataset.

**Fig. 5:**
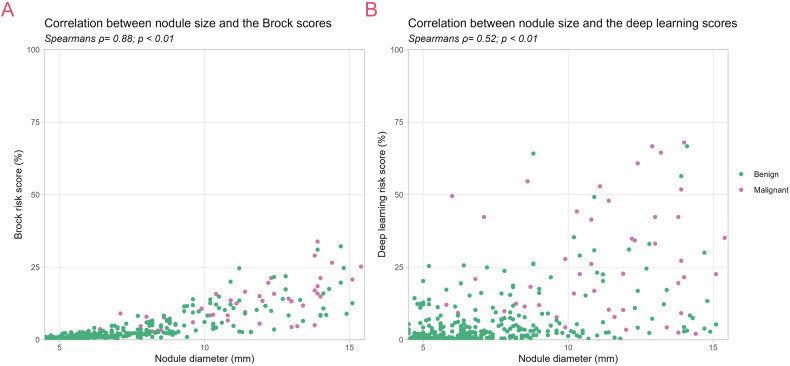
Correlation between nodule size and the malignancy risk scores of the (**A**) deep learning and (**B**) Brock models

Figure 5 showed that DL risk scores were moderately correlated with nodule size (Spearman’s ρ: 0.52; *p* < 0.01), whereas Brock risk scores were highly correlated with nodule size (Spearman’s ρ: 0.88; *p* < 0.01).

**Fig. 6:**
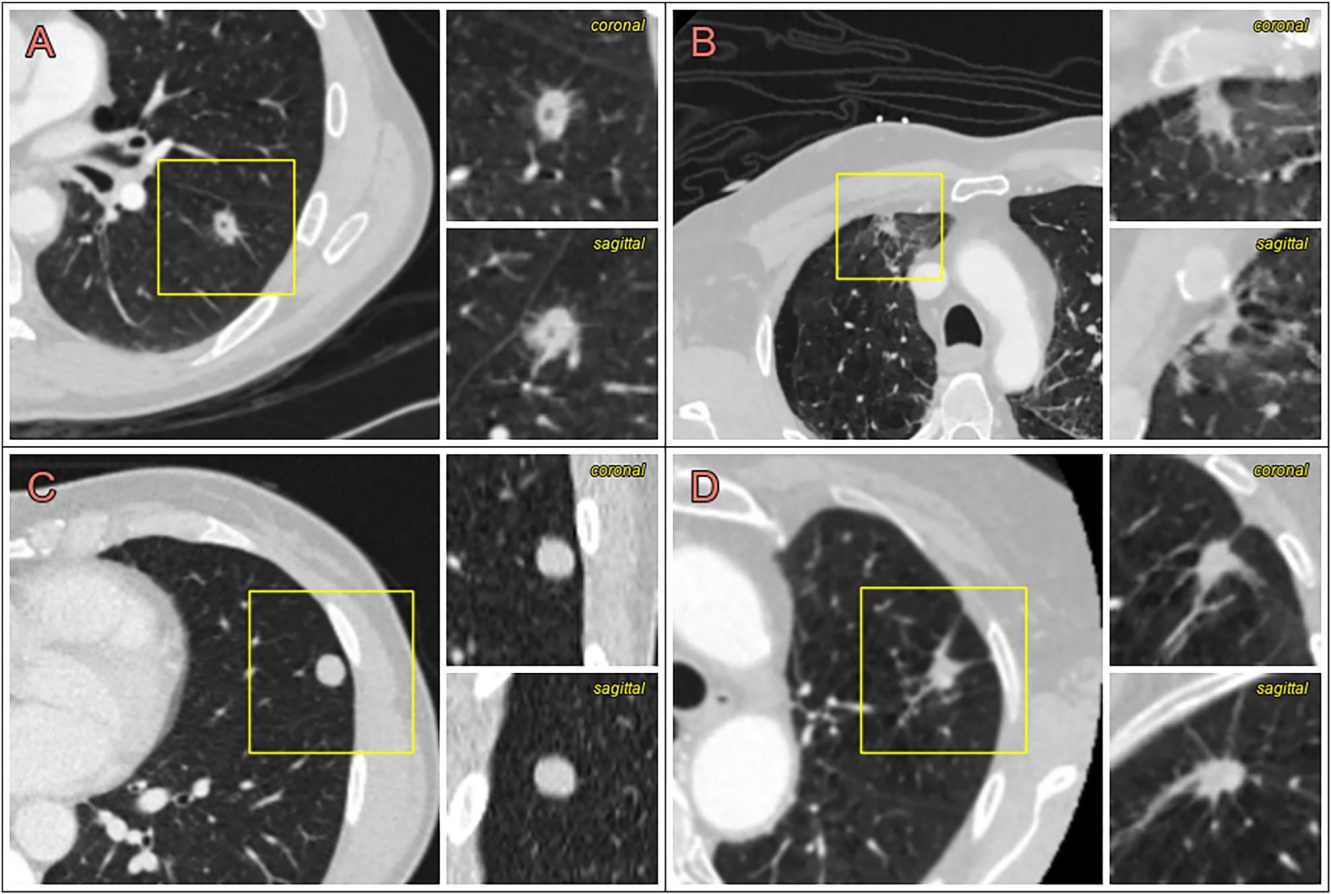
Examples of nodules in the dataset and their risk scores: **A** malignant nodule with a high DL score (65%) and low Brock score (5%) in a 64-year-old man with three other nodules. **B** Benign nodule with a low DL score (3%) and high Brock score (16%) in a 61-year-old man, with no other nodules. **C** Benign nodule with a low DL risk score (0.3%) and high Brock score (10%) in a 45-year-old woman with one other nodule. **D** Benign nodule with a high DL risk score (17%) and high Brock score (21%) in an 81-year-old woman

Figure 6 shows examples of nodules in the dataset and their risk scores.

## Discussion

In this study, we evaluated the performance of an in-house developed DL model, initially trained and validated on lung cancer screening data, in estimating malignancy risk for incidentally detected pulmonary nodules using clinical routine care data. Our primary objective was to determine the model’s applicability in a clinical care setting and to compare its performance with the established Brock model.

We found that the DL model performed well in predicting malignancy risk and stratifying nodules for incidental nodules in a clinical setting. While the DL model performed equally to the Brock model in the complete dataset (AUC: 0.89; and 0.86; *p* = 0.27, respectively), it outperformed the Brock model in the size-matched subset (AUC: 0.78; and 0.58; *p* < 0.01, respectively). The DL model achieved better sensitivity and specificity than the Brock model when varying thresholds. Both the DL and Brock models showed a performance drop in the more challenging size-matched dataset. Notably, the performance drop was larger for the Brock model than for the DL model, as nodule size is a major discriminating factor for the Brock model. This, together with the weaker correlation of the DL model with nodule size compared to the Brock model, indicates that the DL model incorporates morphological criteria other than location and nodule size alone. DL models are prone to the ‘black box phenomenon’, as is the one presented in this study, making it challenging to determine precisely what drives predictions. Recent studies have investigated the explainability of DL models, which could support clinical interpretability. Even though this research topic is relevant and important for clinical integration, the investigation into explainability methods falls outside the scope of this publication.

The performance of the DL model on data from lung cancer screening has previously been assessed and compared to the Brock model, achieving slightly higher AUCs (0.93 and 0.90, respectively) than in the current study’s incidental data [11]. However, the current study only assessed nodules between 5 mm and 15 mm, and the study by Venkadesh et al also included nodules smaller than 5 mm and larger than 15 mm, making a direct head-to-head comparison difficult.

Previous studies showed a comparable performance when testing a DL algorithm originally developed for a screening setting in a clinical context [10, 12]. Both studies tested the same algorithm, but only the study by Baldwin et al [12] included the same nodule size as the current study (5–15 mm). This study showed equal performance. However, studies in a clinical context are limited, and more evidence is needed.

The relatively good performance of the DL model in a clinical setting is remarkable, given that the conditions are much more variable in a clinical setting compared to a screening setting. CT images were obtained at different (mostly higher) dose levels and variably with or without contrast injection. Though we excluded patients with advanced fibrosis, clinical CT scans are characterised by a variable degree of airway disease or parenchymal disease (e.g. emphysema, postinfectious changes). Future studies need to evaluate the impact of image quality and comorbidities on performance and thus the reliability of DL as a function of certain clinical scenarios.

Moreover, we assessed the calibration of the DL risk scores in clinical routine care data, which showed an overestimation of malignancy risk, indicating the need for recalibration. The peak observed at a predicted probability of about 45% likely represents an artefact due to the small size of the dataset. The observed overestimation could be attributed to the difference in settings: in a high-risk screening population, larger nodules are more likely to be malignant, whereas in clinical practice, similarly sized nodules may often be infectious. Due to the different spectrum of pulmonary comorbidities and clinical indications, morphology is less discriminative in a clinical setting.

This study has several limitations. Firstly, this study only evaluated the standalone performance of the DL model as a first step. Some studies have already shown the potential of AI tools for increasing the performance of human observers [15, 16], but we cannot assume that observers would equally benefit from AI tools in a clinical setting. Secondly, we used the full Brock model without spiculation, as spiculation has a low inter-reader agreement [14]. The full Brock model (2b) is reported to perform equal to or better than the parsimonious model (1b) [17–20]. Some studies have shown that the model with and without spiculation does not differ in performance [8, 21], however, the number of studies is limited. Choosing the full model without spiculation led to risk scores taking more variables into account than the parsimonious model, while leaving the variability low by excluding spiculation. Lastly, calibration plots are influenced by incidence. Although the resampling method used to simulate the target incidence allowed us to assess potential calibration issues, it exaggerates any existing selection bias. Therefore, future studies should validate calibration in a larger representative clinical cohort.

In summary, this study showed a promising standalone performance of the DL model in a clinical setting as a first step, thereby showing its potential for optimising nodule management. It also indicates that adjusting the threshold is necessary to optimally adapt the performance of the DL model to the different clinical conditions. Future studies need to assess the optimal thresholds and evaluate the interaction between radiologists and AI regarding performance and reading time.

## Supplementary information

ELECTRONIC SUPPLEMENTARY MATERIAL

## Abbreviations

AI: Artificial intelligence
AUC: Area under the ROC curve
BTS: British Thoracic Society
CI: Confidence interval
CT: Computed tomography
DL: Deep learning
NCR: Netherlands Cancer Registry
NLP: Natural language processing
ROC: Receiver operating characteristic
