# Supplementary material for: Performance of a screening-trained DL model for pulmonary nodule malignancy estimation of incidental clinical nodules
Source: Eur Radiol. 2025 Jul 15;36(1):85–95. doi: 10.1007/s00330-025-11829-1 (PMC12711984; doi:10.1007/s00330-025-11829-1)
Supplement: Supplementary file 1 — ELECTRONIC SUPPLEMENTARY MATERIAL [file 330_2025_11829_MOESM1_ESM.pdf]

Performance of a screening-trained DL model for pulmonary nodule malignancy estimation of incidental clinical nodules

ELECTRONIC SUPPLEMENTARY MATERIAL

Table A1. Lung cancer incidence for nodules sized 5-15 mm in two Dutch hospitals.

**Year**	**Patients with new positive chest CT scan (n)**	**Patients with new positive chest CT scan and subsequent stage I lung cancer diagnosis within 2 years (n)**	**Lung cancer incidence among indeterminate nodules (%)**
2010	395	3	0.8
2011	465	6	1.3
2012	539	7	1.3
2013	756	10	1.3
2014	842	6	0.7
2015	954	9	0.9
2016	1088	14	1.3
2017	1262	13	1.0
Total	6301	68	1.1

Note **–** Analysis performed using methods described in Hendrix 2023 Table 4 for nodules sized 5-15 mm.

Table A2. CT scanner models and reconstruction kernels.

CT characteristics	
Model	
Toshiba: Aquilion	26 (10)
Toshiba: Aquilion ONE	102 (41)
Toshiba: Aquilion Precision	30 (12)
Siemens: Biograph 40	8 (3)
Siemens: Sensation 10	1 (0.4)
Siemens: Sensation 16	35 (14)
Siemens: Sensation 64	24 (10)
Siemens: Somatom Definition AS	7 (3)
Siemens: Somatom Definition AS+	2 (1)
Siemens: Somatom Definition Flash	4 (2)
Philips: Brilliance 40	1 (0.4)
Philips: Brilliance 64	5 (2)
Philips: Gemini TF TOF 64	1 (0.4)
Philips: iCT 256	4 (2)
GE medical systems: LightSpeed VCT	1 (0.4)
Kernel	
Toshiba: BODY	2 (1)
Toshiba: FC01	1 (0.4)
Toshiba: FC03	2 (1)
Toshiba: FC07	1 (0.4)
Toshiba: FC09	40 (16)
Toshiba: FC09-H	4 (2)
Toshiba: FC18	7 (3)
Toshiba: FC30	1 (0.4)
Toshiba: FC52	4 (2)
Toshiba: FC83	14 (6)
Toshiba: FC86	82 (33)
Siemens: B30f	30 (12)
Siemens: B31f	1 (0.4)
Siemens: B40f	1 (0.4)
Siemens: B41f	22 (9)
Siemens: B60f	10 (4)
Siemens: B70f	5 (2)
Siemens: I30f	10 (4)
Siemens: I31f	1 (0.4)
Siemens: I70f	1 (0.4)
Philips: B	2 (1)
Philips: C	2 (1)

Philips: IMR1, Soft Tissue	2 (1)
Philips: L	5 (2.0)
GE medical systems: STANDARD	1 (0.4)

Note – Data are number of CT studies with percentages in parentheses
